# Medical Miscellany

**Published:** 1919-03

**Authors:** 


### MEDICAL MISCELLANY

### Sanitation of Hospitals, Poor Houses and Jails.

The Texas State Health Department is sending out the following instructions with reference to hospitals, poor houses and jails. County and city physicians and all authorities should adhere to the advice as though it were mandatory, and every physician should use his influence to see that the health regulations as to these places are strictly followed:

Sanitation is defined as the establishment of conditions favorable to health—the abatement of menaces and preparation against disease. Pure air, good lighting, uniform heating, safe water, proper disposal of sewage and liquid wastes, wholesome food and general cleanliness of persons, buildings and grounds are all essentials which should be provided.

Floors: Floors of buildings should be not less then 18 inches above the surface of the soil, and the entire space under such floors should be kept dry, clean and free from any accumulation of rubbish, debris and filth. Such space under the floors should be enclosed and provided with screened openings to secure adequate ventilation. It is important that all floors be made ratproof and verminproof and openings through the floor or walls for chimneys, plumbing, water pipes or other purposes shall also be closed tight against the entry of rats and vermin.

Basement Rooms: Basement rooms should never be used for living or sleeping quarters unless they are well lighted and ventilated, the ceiling being not less than seven feet above the adjoining ground level, and the floors and that portion of the walls below the ground level must be made waterproof and dampproof.

Lighting and Ventilation: The window area of each room, should be at least one-tenth of the floor space. Windows provided with movable sash can be raised and lowered as required to secure proper ventilation. 600 cubic feet of air space should be provided for each person occupying a room or hall.

Heating: It is highly desirable that as uniform a degree of temperature be obtained during the winter months as is possible. 68° to 70° Fahr. is probably the most desirable temperature with relative humidity of 50 per cent.

Screening: All sleeping apartments, dining rooms, or other rooms where food may be prepared or eaten should be screened with not larger than 16-wire mesh to effectively prevent the entry of flies, mosquitoes and other insects.

Water Supply: Should any doubt exist as to the quality of water being obtained, samples should be sent to the State Board of Health for examination. Cistern water should only be used when properly collected and stored in a well-protected reservoir. Same should be screened with not less than 16-wire mesh against the entry of mosquitoes. Surface wells are often subject to contamination unless special precautions are taken for their protection from surface and sub-surface drainage from barns, privies, cesspools, garbage piles and other sources of contamination.

Sewerage: Where possible, all water closets, sinks, bath tubs, slop hoppers, or similar plumbing fixtures should be connected to a sanitary sewer system. They should at all times be kept clean, sanitary and in good working order. The floors and walls around such fixtures should be constructed or covered with some inpervious material and scrubbed daily with soap and water. Cesspools are not to be recommended as a means of disposal because of danger to surrounding water supplies. If it is not possible to secure sanitary sewerage facilities some type of approved privy should be adopted and maintained.

Drainage: The site of all buildings should be so graded as to readily carry off any down-spout or surface drainage, and not allow it to stand in the vicinity of the building.

Garbage: Suitable metal receptacles .with tight fitting covers should be provided for garbage, refuse, and rubbish and the contents frequently removed and destroyed by burning. The entire premises should at all times be kept free from accumulations of filth, ashes, garbage, etc.

Spitting: Spitting on floors, walls, or steps of all buildings should be prohibited. Wide mouthed cuspidors containing formaldehyde or carbolic solutions, should be provided in sufficient numbers for public convenience and they much be cleansed thoroughly every day.

Sweeping: Sweeping should be performed daily in the most dustless manner possible and at an hour when the building if free of occupants. Good sweeping compounds may be secured, or sawdust dampened with a 2 per cent. absolute formaldehyde or 5 per cent. carbolic solution will accomplish the purpose. A cloth moistened with a 2 per cent. absolute formaldehyde or other equally efficient disinfectant should be used for dusting. Burlap, coco, Japanese or Chinese matting, or other absorbant covering should not be used on floors of assembly halls, dining rooms, halls, or stairways.

Fumigation: Every three or four months every public building should be fumigated with formaldehyde-parmangenate of potash mixture. Every cell or room occupied by any person suffering with a communicable disease should be promptly disinfected. Fumigation, however, cannot be expected to take the place of washing walls and floors with soap and water, which should be done frequently. County Health Officers should be advised with for specific information on this subject.

Common Drinking Cup: The common drinking cup is condemned in any and all cases. Drinking fountains should be provided. These can be secured with or without running water.

Roller Towels: No roller or public towels should be permitted.

Bedding: Every part of every bed, including the matress, sheets, blankets and bedding, should be kept in a clean, dry and sanitary condition, free from filth, urine or other foul matter in or upon same. It should also be kept free from the infection of lice, bed bugs, or other insects, being sterilized as frequently as is necessary to maintain these conditions.

Bathing: All inmates should be required to take a bath at least once each week, unless liable to injury thereby, and should be provided with soap and towels therefor.

Isolation: All inmates suffering from communicable diseases and also all insane patients should be isolated immediately.

Food: Only good, wholesome, well cooked food should be furnished.

---

### Church Organizes Medical Board to Supervise Health of Its Missionaries.

A medical department, under the direction of the Board of Foreign Missions, to guard the health efficiency of its missionary workers, has been established by the Methodist Episcopal Church in connection with its missionary centenary to raise $120,000,000—$85,000,000 for the Church North and $35,000,-000 for the Church South—for the general world upbuilding and the extension of its missionary work at home and abroad. No other church has organized such a department.

Dr. J. G. Vaughan, M. D., formerly of Nanchang, China, is executive secretary of the new department with temporary offices at the headquarters of the Missionary Centenary, 111 Fifth Avenue, New York. Mr. Vaughan was graduated from the Northwestern Medical School, Chicago, in 1907, and for six years was a medical missionary in China. On his return to this country in 1916 he became connected with the office of the chief surgeon of the Chicago, Rock Island & Pacific Railroad in Chicago. He left that position to organize the new medical department of the Methodist Foreign Missionary Board.

Missionaries in the field and on furlough will have the benefit of counsel from the new department, while all candidates will undergo their medical examinations from the physicians in charge.

To provide for the best service in this respect, suitable offices and equipment will be obtained, with a sufficient staff of trained workers to meet the increasing demands arising from the enlarging force which the Centenary program will require in the field. The Church invests from $20,000 to $50,000 in each missionary for life work and it will be one of the duties of the medical department to see that each person accepted is a "good risk." Supervision of the health of the workers in the field will gradually be taken over by the new department.

### Making Over Faces.

If the Boston Red Cross nurse, who was a sculptor before going overseas, can do all she is said to do in the way of making artificial parts of the face that can not be told from the genuine flesh and blood, face wounds will not be regarded with such horror in future. The surgeons have long been able to handle most wounds of the face successfully, but often disfiguring scars have been left or destroyed parts have been unsightly. The process, by which the wounded face is made to look as good as new, is to take a plaster cast, fill the missing portions in with plaster, and then by a process of electrolysis, construct these portions of copper. These copper parts, noses, ears, or chins, can be enameled so realistically and attached so skillfully that they cannot be detected.

